# Large‐scale proteomics analysis of Alzheimer's Disease plasma blood reveals sex and APOE specific signatures

**DOI:** 10.1002/alz70856_106951

**Published:** 2026-01-12

**Authors:** Simona Merlini, Roberta Diaz Brinton, Francesca Vitali

**Affiliations:** ^1^ University of Arizona, Tucson, AZ, USA

## Abstract

**Background:**

Age, sex, and APOE4 genotype are key non‐modifiable risk factors for Alzheimer's disease (AD), with APOE4 significantly increasing risk, especially in women. The APOE‐sex (APOE‐SX) interaction underscores the need for personalized AD treatments. We analyzed plasma blood proteomic data from the UK Biobank to identify sex and APOE‐specific protein and pathway signatures.

**Method:**

Plasma samples were analyzed for AD‐diagnosed participants and controls. After propensity‐score matching on age and education level, 199 AD cases (133 F) and 199 controls (104 F) from APOE3/3, 3/4 and 4/4 individuals were retained. Protein levels were normalized using z‐scores and a two‐step approach of within‐batch and across‐batches intensity normalization. For each APOE‐SX condition, differentially expressed proteins (DEPs, *p*‐value < 0.05) between AD and controls were identified using linear regression with empirical Bayes estimators to calibrate per‐protein variance using information from all proteins. Gene Set Enrichment Analysis (GSEA) was subsequently conducted using Gene Ontology Biological Processes (GO‐BP) accounting for DEP fold change. Redundant GO‐BP terms (adjusted *p*‐values<0.05) were removed (GO‐BP semantic similarity cut‐off of 0.6). Comparison analyses were then conducted to identify common and unique DEPs and enriched GO‐BPs across APOE‐SX conditions. To achieve this, heatmaps were generated to visualize the overlap and differences in protein expression and pathway enrichment across groups, enabling systematic between‐group comparisons.

**Result:**

Differential expression analysis identified 95 common DEPs in females and 7 DEPs among APOE4 carriers, while no GO‐BPs were shared across all sex‐genotype subgroups. Female APOE4 carriers exhibited the highest number of DEPs (F4/4: *n* = 759; F3/4: *n* = 698) while male APOE3 carriers had the lowest (*n* = 102), but the number of enriched GO‐BPs was comparable to across APOE‐SX conditions, suggesting that a greater number of dysregulated proteins are involved in a limited set of biological processes. GO‐BPs were predominantly downregulated in females and upregulated in males. Notably, humoral immune response mediated by circulating immunoglobulin was uniquely upregulated in F4/4, whereas interferon‐gamma response was a shared pathway in males across APOE genotypes.

**Conclusion:**

These findings suggest APOE‐SX specific proteomic signatures and altered biological processes in AD, highlight strong sex differences in AD plasma proteomics and support the development of personalized therapeutics considering APOE‐SX interaction.

